# Aqueous Extract of Leaves and Flowers of *Acmella caulirhiza* Reduces the Proliferation of Cancer Cells by Underexpressing Some Genes and Activating Caspase-3

**DOI:** 10.1155/2024/3293305

**Published:** 2024-02-10

**Authors:** Huiny Miriane Tienoue Fotso, Mary-Ann Mbong Angie, Françoise Raïssa Ntentie, Inelle Makamwe, Ferdinand Lanvin Edoun Ebouel, Emmerencia Kenjing Ndansack, Enyong Julius Oben

**Affiliations:** ^1^Department of Biochemistry, Faculty of Sciences, University of Yaounde 1, P.O. Box: 812, Yaounde, Cameroon; ^2^Department of Biological Science, Higher Teachers' Training College, University of Yaounde 1, P.O. Box: 47, Yaounde, Cameroon; ^3^Centre for Food and Nutrition Research, Institute of Medical Research and Medicinal Plant Studies, MINRESI, P.O. Box: 13033, Yaounde, Cameroon; ^4^Cameroon Nutrition and Dietetics Research Center, J&A Oben Foundation, P.O. Box: 8348, Yaounde, Cameroon

## Abstract

The increasing prevalence of cancers and the multiple side effects of cancer treatments have led researchers to constantly search for plants containing bioactive compounds with cell death properties. This work aimed at evaluating the antiproliferative effect of an *Acmella caulirhiza* extract. After evaluation of the *in vitro* antioxidant potential of the three extracts of *Acmella caulirhiza* (aqueous (AE-Ac), hydroethanolic (HEE-Ac), and ethanolic (EE-Ac)) through the scavenging of DPPH^●^ and NO^●^ radicals, the extract with the best antioxidant activity was selected for bioactive compound assessment and antiproliferative tests. Subsequently, the cytotoxic activity was evaluated on the viability of breast (MCF-7), brain (CT2A, SB-28, and GL-261), colon (MC-38), and skin (YUMM 1.7 and B16-F1) cancer lines using the MTT method. Then, the line where the extract was the most active was selected to evaluate the expression of certain genes involved in cancerogenesis by RT-PCR and the expression of cleaved caspase-3 involved in cell death mechanism by western blot. The AE-Ac showed the best scavenging activity with IC_50_s of 0.52 and 0.02 for DPPH^●^ and NO^●^, respectively. This AE-Ac was found to contain alkaloids, flavonoids, and tannins and was more active on YUMM 1.7 cells (IC_50_ = 149.42 and 31.99 *μ*g/mL for 24 and 48 h, respectively). Results also showed that AE-Ac downregulated the expression of inflammation (IL-1b (*p* = 0.017) and IL-6 (*p* = 0.028)), growth factors (PDGF (*p* = 0.039), IGF (*p* = 0.034), E_2_F_1_(*p* = 0.038), and E_2_F_2_(*p* = 0.016)), and antiapoptotic protein genes (Bcl-2 (*p* = 0.028) and Bcl-6 (*p* = 0.039)). The cleaved caspase-3 was positively modulated by the AE-Ac inducing thus cell death by apoptosis. AE-Ac showed inhibitory effects on the expression of genes involved in cancer progression making it a potential health intervention agent that can be exploited in cancer therapy protocols.

## 1. Introduction

Despite various technological advances, the cancer survival rate is still very low and is associated with about 9.96 million deaths worldwide [1]. Breast cancer is the most common cancer with an incidence of 11.7%, followed by lung (11.4%), colorectal (10%), prostate (7.3%), stomach (5.64%), and other types of cancer (53.9%) [1]. The word “cancer” refers to a group of diseases that involve abnormal cell growth and invasion of adjacent or distant cells (or tissues). Various agents, both exogenous (radiation, viruses, or toxins) and endogenous (mutations), can affect the cell at several levels (genetic, biochemical, and epigenetic) and directly cause the deregulation of programmed cell death and initiate carcinogenesis [2].

Initiation of extrinsic apoptosis cell death pathway occurs when death receptors (Fas), tumour necrosis factor (TNF-*α*) receptors (TNFR1 and TNFR2), and TNF-related apoptosis-inducing ligand (TRAIL) receptors DR4 and DR5 are occupied by their respective ligands [3, 4]. The intracellular portions of death receptors possess a conserved protein-protein interaction domain known as the death domain, which is a binding site for adaptor proteins, such as the TNF receptor-associated death domain (TRADD) and the Fas-associated death domain (FADD), as well as initiator caspase 8 [5]. Activated caspase-8 in turn stimulates effector caspase-7, enabling cleavage of the death agonist protein BH3, which translocates to the mitochondria and triggers cytochrome C release. In the cytosol, cytochrome C forms a multiprotein complex structure with apoptotic protease activating factor-1 (Apaf-1) and procaspase-9 so-called apoptosome. The apoptosome permits the conversion of procaspase-9 to active caspase-9, which in turn contributes to the activation of effector caspase signaling, destroying the cell through apoptosis [5, 6]. In contrast to antiapoptotic proteins (Bcl-2, Bcl-6, etc.), proapoptotic proteins (Bcl-2 family) act by forming pores in the mitochondrial membrane to release cytochrome C [7].

Once cancer is initiated, the cells immediately begin to secrete several factors such as vascular endothelial growth factor (VEGF), transformative growth factor (TGF), metalloproteinase 2 and angiopoietin-1 (Ang-1) that promote the formation of the new vessels which permit cells to have nutrients, blood, and energy and thus escape chemotherapies [8].

The anticancer treatments target several mechanisms through the use of antimetabolites (raltitrexed), alkylating agents (cyclophosphamide), topoisomerase inhibitors (doxorubicin), mitotic spindle poisons (vincristine), and cytotoxic agents (bortezomib), but all are accompanied by severe side effects (relapses, severe anaemia, and weight and hair losses). Researchers believed that the exploitation of the mechanisms of cell death remains a better alternative to cancer management. Thus, several medicinal plants such as *Cola verticillata*, Indonesian cucumber, and even those of the *Amaryllidaceae* family have been shown to have beneficial properties in the management of diseases including cancers [9–11]. These plants exert their activities due to the presence of some bioactive compounds such as alkaloids and phenolic compounds. Alkaloids from the *Amaryllidaceae* family inhibit the independent effects of p53 on the proliferation of colon cancer cells [12]. Phenolic compounds such as ampelopsin and apigenin through their antioxidant and anti-inflammatory properties induce cell death by apoptosis, suppressing miR-512-3p and promoting the G1 phase of cell cycle involving the p27 Kip1 protein in glioma and breast cancer cells [13–16]. Other bioactive compounds like tomentosin, a terpenoid isolated from plants of the *Asteraceae* family such as *Inula viscosa*, and jolkinolide B (extracted from *Euphorbia kansui*) inhibit the proliferation and migratory activity of cancer cells by downregulating the PI3K-Akt pathway and the expression of certain proinflammatory genes [17–19]. Meilawati et al. [20] have shown that scopoletin, a coumarin present in most edible plants, exerts its anticancer activities through multiple mechanisms including the modulation of cell cycle arrest, the induction of apoptosis, and the regulation of multiple signaling pathways. Thus, *Acmella caulirhiza*, a flowering plant belonging to the *Asteraceae* family, is seasonally found in humid tropical areas. It is used as an ornamental plant but is also consumed as a vegetable by the people of Madagascar and Comoro Islands [21]. Traditionally, the whole plant is used to fight respiratory diseases (cold, asthma, and tuberculosis), baby diaper rash, dental caries [22], and cancer [23]. Studies also revealed that its crude extracts possess anti-inflammatory, antimicrobial, and antioxidant properties [24] which are commonly noted in plants with proven anticancer potential [18, 25], thus making *Acmella caulirhiza* a potential candidate in the treatment of cancer. Hence, the present study aimed at evaluating the antiproliferative properties of the aqueous extract of *Acmella caulirhiza* (AE-Ac) on some cancer cell lines.

## 2. Materials and Methods

### 2.1. Materials

#### 2.1.1. Cell Lines

The cancer cell lines used for this study as described in Table 1 were obtained from the cell bank of the Rothlin-Ghosh Lab, Howard Hughes Institute, Yale School of Medicine (USA).

#### 2.1.2. Cell Culture

Cells were grown in their respective media at 37°C in a humidified atmosphere with 5% CO_2_ and 95% air. The cells were trypsinized (0.1% trypsin) at 85% confluency.

#### 2.1.3. Plant Material and Preparation of Extracts

The leaves and flowers of *A*. *caulirhiza* were collected in October 2018 at *Bandjoun* (West Region, Cameroon) and identified at the National Herbarium of Cameroon (NHC) under the number 602 in comparison with specimen number 57420/NHC of the herbarium. The material was then sorted, cleaned, and dried until constant weight before being powdered and stored at room temperature in a tightly closed amber bottle. The extracts were prepared according to the protocol of Fiardilla et al. [26] with slight modifications. For the preparation of the aqueous, ethanolic, and hydro-ethanolic extracts, the same quantity of *Acmella caulirhiza* powder (100 g) was macerated in 1200 mL volume of distilled water (for 24 hours), 95% ethanol (for 72 hours), and a mix of water and 95% ethanol at a ratio of 1 : 1 (for 48 hours), respectively, at room temperature. The supernatant of each mixture was collected by filtration using Whatman paper No. 3. Each resulting filtrate was frozen and then freeze-dried using a USIFROID SMH 45 (Lagep) to obtain the different extracts labelled AE-Ac for aqueous extract, HEE-Ac for hydroethanolic extract, and EE-Ac for ethanolic extract.

### 2.2. Determination of the Antioxidant Potential and Bioactive Compound Content of the Best Extract of *Acmella caulirhiza*

The antioxidant power of *Acmella caulirhiza* extracts was evaluated through its ability to trap nitric oxide (NO) and 1,1-diphenyl-2-picrylhydrazyl radical (DPPH).

#### 2.2.1. Antioxidant Power of Acmella caulirhiza Extracts

- NO radical: The ability of the different extracts to scavenge the NO radical was evaluated through the method of Hossain et al. [27]. To 1 mL of crude plant extract (125, 250, 500, and 1000 *μ*g/mL) dissolved in methanol (0.2%), was added 2 mL of 10 Mm sodium nitroprusside (in phosphate buffer pH 7.4; 50 mM). The mixture was homogenized and incubated at 25°C for 15 minutes. After incubation, 1 mL of sulfanilic acid (0.33% in 20% glacial acetic acid) was added to 0.5 mL of the mixture and incubated for 5 min for complete diazotization. Then, 1 mL of naphthylethylene diamine dihydrochloride (0.1%) was added, and the solution was homogenized and incubated at room temperature for 30 min. A pink chromophore is formed when light is scattered. The absorbance was read at 540 nm against the blank (the control was the reaction mixture without nitroprusside).
- DPPH radical: The evaluation of the DPPH^●^ radical scavenging capacity was done by spectrophotometry through the method of Katalinié et al. [28]. A volume of 50 *μ*L of different concentrations (125, 250, 500, and 1000 *μ*g/mL) of each extract was added to 1.950 mL of freshly prepared DPPH methanolic solution. The mixture was incubated in the dark for 30 min and the absorbance was read at 515 nm against the DPPH reagent blank.

The results were expressed as a percentage of NO^●^ and DPPH^●^ radical scavenging according to the following formula:

(1) $$\begin{array}{c} \%\;\text{of}\;\text{scavenging}=\frac{\text{absorbance}\;\left(\text{control}\right)-\text{absorbance}\;\left(\text{assay}\right)}{\text{absorbance}\;\left(\text{control}\right)}\times 100. \end{array}$$

The extract with the best antioxidant capacity was selected for further work.

#### 2.2.2. Quantitative Phytochemical Analysis

- Estimation of total phenolic content: The phenol content was evaluated using the method described by Singleton and Rossi [29]. To 30 *μ*L of extract (1 mg/mL) prepared in an ethanol solution, 1 mL of Folin–Ciocalteu (0.2N) solution and 1 mL of sodium carbonate were added. Thirty (30) minutes after incubation at 25°C, the absorbance was read at 750 nm. Gallic acid was used as standard and was treated in the same conditions as the extract. The total phenolic content was expressed in microgram gallic acid equivalence/g of dry matter (*μ*g GAE/g DM).
- Estimation of flavonoid content: The flavonoid content was evaluated using the method described by Bohorun et al. [30]. To one mL of the extract (1 mg/mL), 1 mL of aluminum chloride (10%), 1 mL of potassium acetate (1 M), and 5.6 mL of distilled water were added. The mixture was allowed to stand at 25°C for 30 min. The absorbance of the reaction mixture was read at 420 nm with a spectrophotometer. Catechin was used as the standard and treated in the same conditions. The flavonoid content was expressed in microgram catechin equivalence per gram of dry matter (*μ*g CaE/g DM).
- Estimation of alkaloid content: Quantification of the total alkaloid content in the extract was performed according to the method described by Singh et al. [31] with slight modifications. 100 mg of extract was dissolved in 10 mL of ethanol solution (80%, v/v). The mixture was homogenized and centrifuged for 10 min at 4000 g, 1 mL of the supernatant was introduced into a test tube, followed by the addition of 1 mL of acidified FeCl_3_ solution (FeCl_3_, 0.025 M; HCl, 0.5 M) and 1 mL of an ethanolic solution of 1,10-phenanthroline (0.05 M). The mixture was then homogenized and incubated for 30 min at 100°C in a water bath. The absorbance of the reddish complex formed was read at 510 nm against the blank. Quinine (10 *μ*g/mL) was used as standard, and the alkaloid content was expressed in microgram quinine equivalence per gram of dry matter (*μ*g QiE/g DM).
- Estimation of tannin content: The method described by Bainbridge et al. [32] was used to estimate the total tannin content of the AE-Ac. In the protocol, 1 mL of the extract (1 mg/mL) was mixed with 5 mL of working solution (50 g of vanillin +4 mL of HCl in 100 mL of distilled water) and the mixture was incubated at 30°C for 20 min. The absorbance was read at 500 nm against the blank. Gallic acid (0–1000 *μ*g/mL) was used as standard, and the calibration curve was used to compute the tannin content of the extract. The results were expressed in micrograms of gallic acid equivalence per gram of dry matter (µg GAE/g DM).
- Estimation of total terpenoid content: Total terpenoids were determined according to the method of Ghorai et al. [33]. To 500 mg of extract was added 3.5 ml of ice-cold 95% methanol. It was homogenized before centrifugation at 4000 g for 15 min at room temperature and the supernatant was collected. To 200 *μ*l of supernatant, 1.5 ml of chloroform was added, and the mixture was then roughly mixed and left to stand for 3 minutes. Then, 100 *µ*l of sulfuric acid was added and the whole mixture was incubated at room temperature for 2 h in the dark. Then, carefully and gently the supernatant was decanted without disturbing the precipitation. Then, 1.5 ml of 95% methanol was added and vortexed until the precipitation completely dissolved in the methanol and read at 538 nm. Results were expressed using linalool as a reference molecule.

### 2.3. Cell Viability Assay

Cell viability was performed using the thiazolyl blue tetrazolium bromide (MTT) according to Ahmad et al. [34] with some modifications. About 5000 cells of B16F and YUMM 1.7; 10000 of CT2A, MC-38, and SB-28; 15000 of GL231; and 20000 MCF-7 were plated per well in 96-well plates overnight. They were then treated with the best antioxidant capacity extract at 5, 10, 50, 100, 250, or 500 *μ*g/mL and incubated for 24 h or 48 h. MCF-7 cells were also treated for 72 h (based on the fact that its half-life was about 42 hours). At the end of the different incubation periods, the treated media were removed and 10 *μ*L of 5 mg/mL MTT solution was added to each well and then incubated for 4 h. Later, 100 *μ*L of DMSO was added to each well and plates were protected from light for incubation overnight. The absorbance was read at 570 nm.

(2) $$\begin{array}{c} \text{Cell}\;\text{Viability }\left(\%\right)=\left(\frac{\text{Mean}\;\text{OD}}{\text{Control}\;\text{OD}}\;\right)\times 100. \end{array}$$

### 2.4. Effect of the Extract on Lipid Peroxidation

YUMM 1.7 cells were used for this purpose. About 3000 cells were seeded per well in a 96-well plate and incubated overnight (12 h). They were then divided into two series; the first series contained wells treated on one side with aqueous extract of Acmella caulirhiza (at concentrations of 50, 100, and 150 µg/mL) and on the other hand with 0.5 mM ferrostatin-1 (Selleckchem, USA). In the second series of wells, to aqueous extract of *Acmella caulirhiza* was added 4.0 *μ*M RLS3 (Selleckchem, USA). The second series also contained wells treated only with RSL3. Both series contained nontreated cells. The test was done thrice. The plate was afterwards incubated for 4 h. CellTiter-Glo 2.0 Assay kit (Promega Co) was used to evaluate cell viability according to the protocol described by the manufacturer.

### 2.5. Effect of the Extract on the Expression of Some Genes Involved in Cancerogenesis

The YUMM 1.7 cells (350000/well in a 6-well plate) were treated with 150 *μ*g/mL of AE-Ac in triplicate and incubated for 24 h. RNA extraction and purification were carried out for each treatment using the RNeasy Mini Kit (QIAGEN, USA). RNA quantification was done using the NANODROP 2000 Spectrophotometer of Thermo Fisher Scientific. The purified RNA was then used to synthesize cDNA using Bio-Rad's iScript cDNA Synthesis Kit. For qPCR analyses, the KAPA SYBR FAST Universal kit with corresponding primers was prepared for each gene in triplicate. The C1000 Touch Thermal Cycler of Bio-Rad with associated software was used to run qPCRs. All kits were used according to manufacturers' protocols. The genes evaluated included growth factor (PDFG, IGFR-1-R, TGF*β*, VEGF, E2F1, and E2F2), antiapoptotic (Bcl-2 and Bcl-6), and cytokine (TNF-*α*, IL-1b, IL-6, and IL-10) genes. Genes including ribosomal protein 18s, glyceraldehyde-3-phosphate dehydrogenase (GAPDH), and hypoxanthine phosphoribosyltransferase-1 (hprt-1) were used as control genes (Table 2). Ct values for each treatment were quantified according to the 2−∆∆Ct methodology.

(3) $$\begin{array}{c} \text{Gene}\;\text{Expression}=2\times \left[20-\left(\text{Ctsample}-\text{AvCt}\;\text{House Keeping}\;\text{genes}\right)\right],\\ \text{Relative}\;\text{Expression}=\text{Expression}\;\text{of}\;\text{control}-\text{Expression}\;\text{of}\;\text{sample}. \end{array}$$

### 2.6. Effect of the Extract on the Expression of Cleaved Caspase-3

Following a 24 h of treatment of YUMM 1.7 cells (350000 cells per well in a 6-well plate) with 150 *μ*g/mL of extract or 2 *μ*M of etoposide, cells were lysed using RIPA buffer (50 mM Tris-HCl pH 7.4, 150 mM NaCl, 1% NP-40, 0.1% SDS, and 2 mM EDTA). Protein content was determined using the BCA assay kit (Thermo Fisher Scientific) according to the manufacturer's instructions. Samples (nontreated cells and cells treated with either extract or etoposide) were loaded and run in a polyacrylamide gel for 90 min, at 100 V. Separated proteins were then transferred to a PVDF membrane and blocked using Intercept Blocking Buffer (LI-COR). The membrane was then incubated with a primary antibody from rabbit (caspase-3, Cell Signaling Technology) and mouse antibody for *β*-actin (Cell Signaling Technology) for a period of 24 h in a cold room. The membrane was then washed 3 times with PBST (PBS + 0.1% Tween 20) and incubated with the secondary antibody; the donkey antirabbit antibody (cell signalling technology) was conjugated to a chemiluminescent such as IR680 (red) to detect cleaved caspase-3, and the donkey antimouse antibody conjugated to IR800 (green) to reveal β-actin. The plate protected from light was incubated for 1 h and later visualised using the Odyssey system associated with ImageStudio (LI-COR).

### 2.7. Statistical Analysis

Data were expressed as mean ± standard deviation (SD). Statistical analysis was performed using Statistical Package for Social Science (SPSS) software version 20.0 for Windows. One-way analysis of variance (ANOVA) and the least significant difference (LSD) post hoc test were used to compare means between groups. Significance was set at *p* < 0.05. The IC_50_ and SC_50_ values were obtained by linear regression and Microsoft Excel 2016 spreadsheet software was used to plot the graphs.

## 3. Results

### 3.1. Antioxidant Potential of the *Acmella caulirhiza* Extracts and Bioactive Compound Content of the Best Extract

#### 3.1.1. Antioxidant Potential of Extracts of *Acmella caulirhiza*

**Figure 1:**
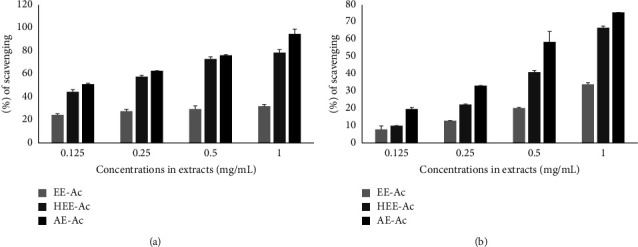
Effect of the different extracts of *Acmella caulirhiza* on NO^●^ (a) and DPPH^●^ (b) radicals.

The NO^●^ and DPPH^●^ radical scavenging potential of the different extracts of *A. caulirhiza* is reported in Figures 1(a) and 1(b). Results showed that the extracts scavenged these radicals in a concentration-dependent manner. The AE-Ac presented the highest scavenging capacity towards NO^●^ and DPPH^●^ with SC_50_ of 0.02 and 0.52, respectively, followed by the HEE-Ac (Table 3). As the tumour microenvironment is characterized by a high oxidative stress state, the AE-Ac with the best antioxidant activity was selected for further study.

#### 3.1.2. Bioactive Content of the Aqueous Extract of *Acmella caulirhiza*

Results revealed the presence of total phenolics, alkaloids, and flavonoids in the aqueous extract of *A. caulirhiza* (Table 4).

### 3.2. Effect of AE-Ac on Cell Viability

**Figure 2:**
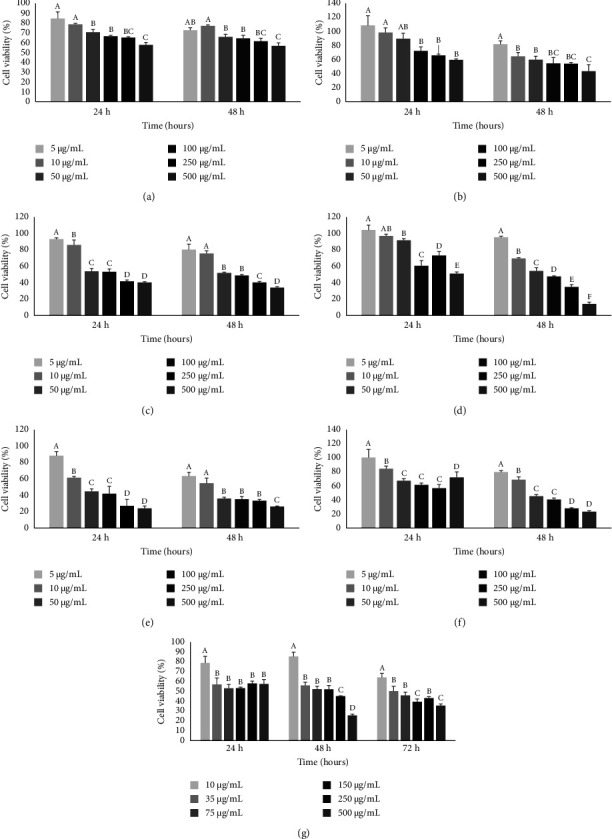
Effect of extract on viability of CT 2A (a), SB 28 (b), GL 261 (c), B16-F1 (d); YUMM 1.7 (e), MC38 (f) and MCF-7 (g) cancer cells. B16-F1: murine melanoma cells; CT2A: nonmetastatic murine glioma (astrocytoma) cells; Gl-261; mouse glioma cells; MC-38: murine colon adenocarcinoma cells; MCF-7 cells: Michigan breast adenocarcinoma Cancer Foundation-7 cells; SB-28: sleeping beauty mouse glioma cells; YUMM 1.7: Yale University mouse melanoma cells. Histograms with different letters are significantly different at *p* < 0.05 for the same period.

A concentration-dependent effect of the AE-Ac was noted on the proliferation of some cell lines as reported in Figures 2(a)–2(g). The CT2A, SB-28, MC-38, and MCF-7 cell lines were the most resistant to the AE-Ac as their IC_50_s were higher than 500 *μ*g/mL after 24 h of exposition. However, after 48 h of treatment, their IC_50_s were >500, 351.48, 145.35, and 215.66, respectively, for CT2A, SB-28, MC-38, and MCF-7 cells. However, the extract caused a significant decrease in the proliferation of GL-261, B16-F1, and YUMM 1.7 cells with respective IC_50_s of 296.81, 487.94, and 149.42 after 24 h and 232.29, 180.26, and 31.99 after 48 h (Table 5). As the antiproliferative properties of the extract were more perceptible on the YUMM 1.7 cell line, it was selected for the evaluation of the extract ability to regulate the expression of certain genes.

### 3.3. Effect of AE-Ac on the Lipid Peroxidation

**Figure 3:**
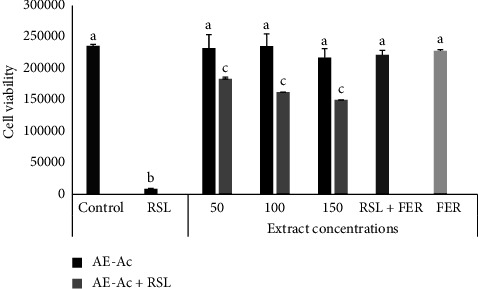
Effect of the extract on lipid peroxidation. Ac: *Acmella caulirhiza*; control: untreated YUMM 1.7 cancer cells; FER: ferrostatin-1 treated cells; RSL: ras small lethal molecule treated cells. Histograms with different letters are significantly different at *p* < 0.05.

The RSL3 induced peroxidation in YUMM 1.7 cells, but the extract significantly limited the peroxidative properties of RSL3 for all three concentrations. Nonetheless, this protective property was lower than that of ferrostatin-1 (*p* < 0.05) (Figure 3).

### 3.4. Effect of the AE-Ac on the Expression of Some Genes Involved in Carcinogenesis of the YUMM 1.7 Cells

#### 3.4.1. Effect of the AE-Ac on Proinflammatory Gene Expression

**Figure 4:**
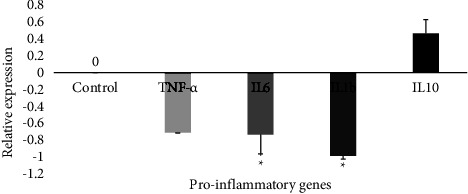
Effect of the extract on inflammatory markers involved in YUMM 1.7 cells. TNF-*α*: tumour necrosis factor-*α*; IL: interleukin. ^*∗*^Significant difference at *p* < 0.05 compared to the control.

Except for IL-10, treatment of YUMM 1.7 cells with the aqueous extract of *A. caulirhiza* led to a decrease in the expression of TNF-*α*, IL-1b (*p* = 0.017), and IL-6 (*p* = 0.028) genes compared to control (Figure 4).

#### 3.4.2. Effect of the Extract on Evaluated Growth Factor Gene Expression in YUMM 1.7 Cells

**Figure 5:**
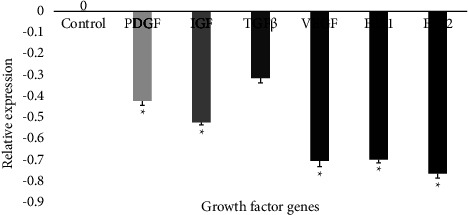
Effect of AE-Ac on certain growth factors in YUMM 1.7 cells. E2F: transcription factors of the E2F family; VEGF: vascular endothelial growth factor; PDGF: platelet-derived growth factor; TGF*β*: transforming growth factor *β*; IGF-1: insulin-like growth factor 1. ^*∗*^Significant difference at *p* < 0.05 compared to the control.

The AE-Ac downregulated the expression of PDGF (*p* = 0.039), IGF-1 (*p* = 0.034), VEGF (0.024), E_2_F_1_(*p* = 0.038), and E_2_F_2_(*p* = 0.016) genes as compared to control (Figure 5).

#### 3.4.3. Effect of AE-Ac on Two Genes of Antiapoptotic Proteins

**Figure 6:**
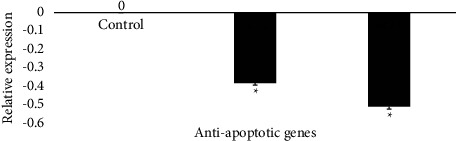
Effect of AE-Ac on some genes of antiapoptotic proteins in YUMM 1.7 cells. Bcl: B cell lymphoma. ^*∗*^Significant difference at *p* < 0.05 compared to the control.

After exposure of YUMM cells to AE-Ac, a significant downregulation of Bcl-2 (*p*=0.028) and Bcl-6 (*p*=0.039) gene expression was observed (Figure 6).

### 3.5. Effect of AE-Ac on the Cleaved Caspase-3's Expression

**Figure 7:**
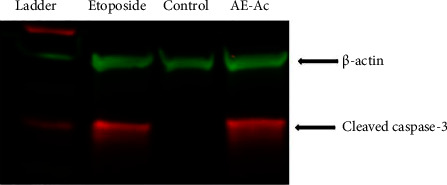
Effect of the AE-Ac on the expression of cleaved caspase-3 in YUMM 1.7 cells.

Results revealed that the AE-Ac induced apoptosis just like etoposide (an inducer of apoptosis) in cells through the activation of cleaved caspase-3, a cysteine protease involved in cell death by nucleic acid degradation (Figure 7).

## 4. Discussion

Cancer cells are unable to control the expression of cell death genes, making them resistant to chemotherapies. Anticancer therapies target several mechanisms of cell death through the use of antimetabolites, alkylating agents, mitotic spindle poisons, and even cytotoxic agents. However, these therapies are toxic to cancer cells and also to normal cells of the body. The control of natural mechanisms of cell death remains a better alternative in the management of cancers. Results of this study which aimed at evaluating the antiproliferative properties of the aqueous extract of *Acmella caulirhiza* revealed that the extract exerted antiproliferative properties through the downregulation of the expression of some genes involved in the cancerogenesis pathway and this was due to the presence of bioactive molecules.

The evaluation of the *in vitro* antioxidant potential (scavenging of DPPH^●^ and NO^●^ radicals) of the three extracts of *Acmella caulirhiza* (EE-Ac, HEE-Ac, and AE-Ac) showed that the AE-Ac exhibited the best free radical scavenging activity (Table 3). This antioxidant capacity of the extract is linked to its high content of phenolic compounds as well as other bioactive compounds like alkaloids (Table 4). Phenolic compounds have free hydroxyl groups and conjugated double bonds in their structures capable of providing hydrogen or electron to a free radical or a metal [35]. Also, it is well known that the bioactive compounds of plants have already demonstrated anticancerous properties in both *in vitro* and *in vivo* studies [11, 36, 37], thus the selection of the aqueous extract for antiproliferative tests.

One of the biological characteristics of cancer cells is the production of reactive oxygen species. One of the major drawbacks of chemotherapy is lipid peroxidation, which is frequently caused by interactions of these species with polyunsaturated fatty acids in lipid membranes. Lipid peroxidation is considered a key biochemical process in the toxicity process that causes cell death as well as oxidative damage to cellular components. During this process, free radicals steal electrons from cell membrane lipids, which jeopardize cell life by causing decreased membrane fluidity, increased membrane permeability, and decreased physiological performance [38]. In this study, the potential of AE-Ac to inhibit lipid peroxidation was measured by evaluating the viability of YUMM 1.7 line cancer cells exposed to RSL3 (a lipid peroxidation activator), which acts by inhibiting glutathione peroxidase 4. AE-Ac at all concentrations (50, 100, and 150 *μ*g/mL) showed protective effects of cell membranes against RSL3 by protecting cells from the peroxidative action of RSL3. This activity could be explained by the antioxidant properties of the aqueous extract of Ac through its ability to scavenge free radicals generated by tumour cells, thus preventing the peroxidation of lipid membrane [38].

The evaluation of the cytotoxic properties of this aqueous extract on breast, brain, skin, and colon cancer cell lines showed that the extract was more active on YUMM 1.7 cells with IC_50_s of 149.42 and 31.99 after 24 and 48 h, respectively (Table 5). Xie et al. demonstrated that alkaloids, flavonoids, coumarins, and terpenoids also present in our extract upregulated the expression of Kip/p27 leading to a decrease in cyclin-D, cyclin-E, and CDK2/4/6 proteins in the melanoma cancer cell lines (WM1361B and WM983A) [16], colon cancer cell lines (HCT-116, LoVo, and DLD-1) [12], and human breast cancer cell line (MCF-7) [37]. This could lead to the breakage of retinoblastoma and E2F proteins, stopping the cell cycle in the G1/G0 phase.

This downregulated the transcription factors NF-*κ*B and upregulated tumour suppressor factors (p53 and p21t) which are cellular gatekeepers of growth.

Cleaved caspase-3 is well known as an executor protease of cell death by apoptosis through the reduction of mitochondrial membrane potential. The AE-Ac induced expression of this protein (Figure 7). The flavonoids in the extract could reduce mitochondrial membrane potential, leading to the release of apoptogenic factors such as Arts, Diablo, Second Mitochondria-Derived Activator of Caspase (SMAC), and High-Temperature Requirement Protein A2 (Omi/HTRA2). These proteins can block the action of the apoptosis inhibitor proteins Bcl-2 and Bcl-xL (which inhibit apoptosis by forming macropores on the mitochondrial membrane and prevent the release of cyt c and the formation of the apoptosome) and the activation of caspase-3 [39, 40]. Once activated, caspase-3 translocates to the nucleus in its cleaved caspase-3 form where it causes cell death by DNA fragmentation and degradation of nucleic acids [6, 41]. Indeed, Bernard et al. have shown that cleaved caspase induced gene transcription by interacting with their promoters and inhibiting their expression within the VEGFA gene in the chip experiment [42, 43]. This resulted in the downregulation of several pathways of angiogenesis (FAS, TRAIL, IFN-*γ*, TNF receptor, and RAC1) in MCF-7 and human Jurkat leukaemia cells [40, 44]. In addition, studies had shown that apigenin (a flavonoid) exerted antiproliferative activities on human melanoma cell lines A375P and A375SM through the activation of the apoptotic pathway and the decrease of the antiapoptotic protein Bcl-2 expression [15, 45].

To escape cell death, cancer cells also alter the expression of genes and proteins associated to inflammation. Some cytokines and proinflammatory proteins like TNF-*α* and IL are promoters of carcinogenesis through the activation of several signaling pathways [3, 7, 46]. PCR analyses showed that treatment of YUMM 1.7 cells with the AE-Ac downregulated the expression of the proinflammatory gene (TNF-*α*, IL-1*β*, and IL-6) (Figure 4). Mathieu et al. demonstrated that alkaloids such as those of *Amaryllidaceae* (lycorine, narciclasine, and haemanthamine) exert anticancer activities through the inhibition of NFkB (an oncogene involved in tumorigenesis and resistance to apoptosis [47]) [12]. It consequently activated the p53 tumour suppressor gene leading to a decrease in the expression of TNF-*α*, IL-6, IL-1*β*, and VEGF in colorectal cancer cells [12]. On the other hand, growth factors (IGF-1, TGFβ, VEGF...) are activated by binding to their respective intracellular receptors, which can activate several signaling pathways involved in cell proliferation [8]. PCR results showed that growth factors' gene expression was downregulated after 4 h of treatment (Figure 5). This would be due to the presence of flavonoids which can prevent the binding of ligands to their membrane receptor such as epidermal growth factor receptor/mitogen-activated protein kinase (EGFR/MAPK), thus inhibiting their activity with the consequence of stopping the cell proliferation [48, 49].

The deregulation of B cell lymphoma family proteins is the main feature of malignant diseases. It is responsible for resistance to cell death and thus to treatment [7, 50]. In this study, exposure of cells to EA-Ac resulted in a downregulation of the expression of antiapoptotic genes Bcl-2 and Bcl-6 (Figure 6) due to the capacity of the terpenoids present in the AE-Ac. Indeed, Yang et al. had shown that borneol, a bicyclic monoterpenoid, caused a significant release of cyt c which promotes apoptosome formation by aggregating caspase-9 with Apaf-1 in the cytosol triggering apoptosis [16, 18]. Results of western blot analysis (Figure 7) show that the extract also induced death by apoptosis through its ability to promote activation of caspase-3.

## 5. Conclusion

The AE-Ac exhibited the highest antiradical scavenging activities due to the presence of bioactive compounds (alkaloids, flavonoids, tannins, and terpenoids). This extract was more cytotoxic on YUMM 1.7 cells after 24 and 48 h incubation periods compared to the other cancer cell lines. The AE-Ac can induce cell death through the underexpression of inflammation, growth factors, and antiapoptotic protein genes. The presence of cleaved caspase-3 after treatment of YUMM 1.7 cells with the extract confirmed its capacity to induce apoptosis. Studies of other antiproliferative pathways of this extract can reinforce the knowledge of the aqueous extract of *Acmella caulirhiza* as a potential candidate for cancer treatment [51].

## Figures and Tables

**Table 1:** Cell lines and description.

Adherent cell lines	Description
MCF-7	Breast adenocarcinoma cells
MC-38	Colon adenocarcinoma cells
B16-F1	Melanoma cells
YUMM 1.7	Melanoma cells
GL-261	Mouse glioma cells
SB-28	Mouse glioma cells
CT2A	Mouse glioma cells

**Table 2:** Primers of the genes used (Sigma-Aldrich DNA oligos template).

Name	Code	Scale	Forward sequence (5′ ⟶ 3′)	Reverse sequence (5′ ⟶ 3′)
TNF-*α*	STD	0.025	GGTGCCTATGTCTCAGCCTCTT	GCCATAGAACTGATGAGAGGGAG
TGFb1	STD	0.025	TGATACGCCTGAGTGGCTGTCT	CACAAGAGCAGTGAGCGCTGAA
IL-6	STD	0.025	TACCACTTCACAAGTCGGAGGC	CTGCAAGTGCATCATCGTTGTTC
IL-1*β*	STD	0.025	CTTGGGCCTCAAAGGAAAGAA	AAGACAAACCGTTTTTCCATCTTC
IL-10	STD	0.025	CGGGAAGACAATAACTGCACCC	CGGTTAGCAGTATGTTGTCCAGC
Bcl-2	STD	0.025	ATGCCTTTGTGGAACTATATGGC	GGTATGCACCCAGAGTGATGC
Bcl-6	STD	0.025	CCTGAGGGAAGGCAATATCA	GTTTAAGTGCAGGGGCCATT
HPRT-1	STD	0.025	GCAGACTTTGCTTTCCTTGG	CGAGAGGTCCTTTTCACCAG
GAPDH	STD	0.025	GCAAGAGAGAGGCCCTCAG	TGTGAGGGAGATGCTCAGTG
IGF-1	STD	0.025	GTGTGGACCGAGGGGCTTTTACT	GCTTCAGTGGGGCACAGTACATC
E2F1	STD	0.025	GATGGTGGGGCTGATATTTG	CAGCGAGGTACTGATGGTCA
E2F2	STD	0.025	ACAACATCCAGTGGGTAGGC	GGGAGCAACTCTGAATGAGC
VEGF	STD	0.025	CACAGCAGATGTGAATGCAG	TTTACACGTCTGCGGATCTT
*β*-Actin	STD	0.025	ACGACGTGGCAGCTCTCGTTGTGG	GGTGCTTCGGTCAGCAGCACGGA
PDGF	STD	0.025	TAGCGCGGAACCTCAGAGAGA	TGGGAGGTCCCCATAGCTCC

E2F: transcription factors of the E2F family; GAPDH: glyceraldehyde-3-phosphate dehydrogenase; HPRT-1: hypoxanthine phosphoribosyltransferase-1 enzyme; IGF-1: insulin-like growth factor 1; Bcl: B cell lymphoma; IL: interleukin; PDGF: platelet-derived growth factor; TGF*β*: transforming growth factor *β*; TNF-*α*: tumour necrosis factor-*α*; VEGF: vascular endothelial growth factor.

**Table 3:** Scavenging concentration 50 of different *Acmella caulirhiza* extracts.

		EE-Ac	HEE-Ac	AE-Ac
SC_50_ (mg/mL)	DPPH^●^	16.69	0.71	0.52
	NO^●^	3.29	0.09	0.02

EE: ethanolic extract; HEE: hydroethanolic extract; AE: aqueous extract; SC_50_: scavenging concentration 50; Ac: *A. caulirhiza*.

**Table 4:** Content of bioactive compounds in the aqueous extract of *Acmella caulirhiza*.

Bioactive compounds	Content (%)
Alkaloids (mg QiE/g DM)	33.23 ± 2.82
Flavonoids (*μ*g QE/g DM)	81.36 ± 19.18
Tannins (*μ*g GAE/g DM)	308.22 ± 28.00
Terpenoids (mg linalool/g DM)	10.06 ± 0.41
Polyphenolic compounds (mg GAE/g DM)	57.53 ± 2.86

Values are expressed as mean ± standard deviation. AE-Ac: aqueous extract of *A. caulirhiza*; *μ*g: microgram; GAE: gallic acid equivalent; QE: quercetin equivalent; QiE: quinine equivalent; DM: dry matter; g: gram.

**Table 5:** Inhibitory concentrations 50 of the AE-Ac on cancer cell lines.

Cell type		CT2A	SB-28	GL-261	B16-F1	YUMM 1.7	MC-38	MCF-7
IC_50_s	24 h	>500	>500	296.81	487.94	149.42	>500	>500
	48 h	>500	351.48	232.29	180.26	31.99	145.35	215.66
	72 h							114.86

B16-F1: murine melanoma cells; CT2A: nonmetastatic murine glioma (astrocytoma) cells; Gl-261; mouse glioma cells; MC-38: murine colon adenocarcinoma cells; MCF-7: Michigan breast adenocarcinoma cancer foundation cells; SB-28: sleeping beauty mouse glioma cells; YUMM 1.7: Yale University mouse melanoma cells.

## Data Availability

The data used to support the findings of the study are available from the corresponding author upon request.

## Abbreviations

AE-Ac: Aqueous extract of *Acmella caulirhiza*
HEE-Ac: Hydroethanolic extract of *Acmella caulirhiza*
EE-Ac: Ethanolic extract of *Acmella caulirhiza*
B16-F1: Murine melanoma cells
BCA: Bicinchoninic acid assay
Bcl: B cell lymphoma
CT2A: Nonmetastatic murine glioma (astrocytoma)
DMSO: Dimethyl sulfoxide
DPPH: 2,2-Diphenyl-1-picrylhydrazyl
EDTA: Ethylenediaminetetraacetic acid
ELISA: Enzyme-linked immunosorbent assay
FER: Ferrostatin
GAPDH: Glyceraldehyde-3-phosphate dehydrogenase
Gl-261: Mouse glioma cells
H: Hours
IC_50_: Inhibitory concentration 50
IL: Interleukin
MC-38: Murine colon adenocarcinoma cells
MCF-7: Michigan Cancer Foundation cells
MTT: 3-(4,5-Dimethylthiazol-2 yl)-2,5-diphenyltetrazolium
NF-*κ*B: Nuclear factor kappa B
NO: Nitric oxide
OD: Optical density
PDGF: Platelet-derived growth factor
PVDF: Polyvinylidene fluoride
RIPA: Radioimmunoprecipitation assay
RSL: Ras small lethal molecule
RT-PCR: Reverse transcription polymerase chain reaction
SB-28: Sleeping beauty cell
SDS: Sodium dodecylsulfate
TBST: Tris-buffered saline with 0.1% Tween® 20
TGF: Transforming growth factor
TNF-*α*: Tumour necrosis factor alpha
YUMM 1.7: Yale University mouse melanoma.
